# The spatial extent of focused attention modulates attentional disengagement

**DOI:** 10.1007/s00426-022-01747-y

**Published:** 2022-10-20

**Authors:** Lisa N. Jefferies, Rebecca Lawrence, Elizabeth Conlon

**Affiliations:** 1 School of Applied Psychology, Griffith University, 7 Parklands Drive, Southport, QLD 4222 Australia; 2 Menzies Health Institute Queensland, Southport, Australia

## Abstract

Attention can be flexibly changed to optimize visual processing: it can be oriented, resized, or even divided. Although much is known about these processes individually, much less is known about how they interact with one another. In the present study we examined how the spatial extent of the attentional focus modulates the efficiency of the first component of attentional orienting, the disengagement of attention. To this end, we used abrupt-onset stimuli of different sizes to trigger the reflexive resizing of the attentional focus (Castiello and Umiltà in Acta Psychol 73:195–209, 1990), combined with a gap task to assess the efficiency of attentional disengagement (Mackeben and Nakayama in Vis Res 33:85–90, 1993). The results of five experiments showed that the magnitude of the gap effect is significantly greater when the scope of attention is small than when it is large, indicating that disengaging attention is delayed when attention is highly focused. Furthermore, these findings highlight that different aspects of attentional control interact with one another, emphasizing the importance of studying them in conjunction.

## Introduction

Our visual environment is rich, complex, and constantly changing. Efficient visual perception, therefore, requires the rapid and flexible processing of relevant information and the filtering out of irrelevant information—a function served by selective visual attention. The focus of attention can be flexibly altered in many ways: it can be rapidly shifted from one object or location to another (e.g., Jonides & Yantis, 1988; Posner, 1990; Posner & Cohen, 1984; Theeuwes, 1992), adjusted in size or spatial extent to encompass larger or smaller objects or regions of space (e.g., Castiello & Umiltà, 1990; Eriksen & Yeh, 1985; Maringelli & Umiltà, 1998), divided into more than one focus (e.g., Awh & Pashler, 2000; Jefferies & Witt, 2019; Jefferies et al., 2022; McMains & Somers, 2004, 2005; Müller et al., 2003), or deployed in alternate forms such as an annulus (e.g., Eimer, 1999, 2000; Jefferies & Di Lollo, 2015, 2017; Juola et al., 1991). Neuroimaging research has shown that attending to specific regions of space results in enhanced activity in areas of the visual cortex that are retinotopically mapped to the attended area (e.g., Müller et al., 2003; Sasaki et al., 2001). It has further been shown that changes to the attentional focus are reflected in changes to neural activity. Functional magnetic resonance imaging research, for example, has shown that as the focus of attention expands or contracts in size, the extent of the activated retinotopic visual cortex also increases or decreases (Müller et al., 2003). Although much is known about the separate processes by which attention can be deployed and changed, relatively little is known about how these processes interact with one another. In the present study, we examine how the spatial extent of the attentional focus interacts with attentional orienting.

Attentional orienting can be automatic, voluntary, or shaped by broader contextual influences (e.g., Awh, Belopolsky & Theeuwes, 2010). When a new stimulus is selected for processing, and orienting is initiated, three independent processes occur: attention is first *disengaged* from the currently attended stimulus, before being *shifted* to the new stimulus, and then finally *engaged* on the new stimulus (Posner, 1980; Posner & Petersen, 1990). Since attention cannot be shifted until it is disengaged, attentional disengagement is essential for successfully interacting with the visual environment. The speed of disengaging attention, however, varies depending on the stimuli, the individual, and the task. For example, the age or anxiety level of an individual affects the efficiency of attentional disengagement (e.g., Greenwood & Parasuraman, 1994; Van der Stichel, 2017), as does whether the stimulus is salient (e.g., Brockmole & Boot, 2009), task-relevant (e.g., Müller et al., 2016), or emotional (e.g., Fox et al., 2002). Here, we consider whether the efficiency of attentional disengagement is also influenced by the size or spatial extent of the attentional focus.

The attentional focus can be expanded or contracted in spatial extent (a process variously referred to in the literature as attentional resizing, focusing, or scaling) to encompass larger or smaller objects or regions of space (e.g., Castiello & Umiltà, 1990; Eriksen & Yeh, 1985; La Berge, 1995). There is evidence, however, of a trade-off between the spatial extent of the attentional focus and the efficiency of processing within the attended region—attentional resources are more densely concentrated within a narrow focus and more diffusely distributed within a broad focus (Eriksen & St. James, 1886; Eriksen & Yeh, 1985). There is physiological evidence to support this trade-off—as the size of the attended region increases, the level of neural activity within the activated retinotopic visual cortex decreases (Müller et al., 2003).

The spatial extent of the attentional focus has been shown to affect many aspects of visual perception. A narrow attentional focus generally leads to faster and more accurate detection and discrimination of stimuli (e.g., Albonico et al., 2017; Castiello & Umiltà, 1990; Eriksen & St. James, 1986; Eriksen & Yeh, 1985; Galera et al., 2005; Lawrence et al., 2020; Maringelli & Umiltà, 1998, Turatto et al., 2000), improves the spatial resolution of visual processing (e.g., Balz & Hock, 1997; Carrasco et al., 2000; Goodhew et al., 2016; Yeshurun & Carrasco, 1998; for a review, see Carrasco, 2011), and influences the earliest stages of attention-modulated brain activity (e.g., Fu et al., 2005; Luo et al., 2001; Müller et al., 2003; Wang et al., 2010).

A narrow focus of attention not only increases the precision with which a stimulus is processed (e.g., Carrasco et al., 2000; Yeshurun & Carrasco, 1998), but also results in the stimulus appearing to be both larger (Kirsch et al., 2018), more salient (Carrasco et al., 2004), and more strongly represented in visual memory (Gmeindl et al., 2020). Any of these factors could cause attention to be disengaged more slowly from the stimulus when the attentional focus is narrow than when it is broad. The present study tests whether attention is disengaged more efficiently when it is narrowly focused by combining two well-established techniques: the gap paradigm, to assess attentional disengagement, and the use of abrupt-onset stimuli, to trigger resizing of the attentional focus.

The gap paradigm (e.g., Mackeben & Nakayama, 1993) allows attentional disengagement to be measured separately from the shifting and engaging components of attentional orienting. In Mackeben and Nakayama’s task, a central fixation cross was displayed at the start of every trial. After a brief delay, a red circle appeared, which cued the location of a subsequent target. An array of distractors (vertical lines) and a target (a vernier-offset line) was then displayed, and the task was to indicate the offset direction of the vernier target. Critically, the fixation cross was removed prior to the onset of the cue (the *Gap* condition) on half of the trials; on the remaining trials, the fixation cross remained present throughout the trial (the *No-Gap* condition).

In the No-Gap condition, two processes were required for attention to orient from the fixation cross to the cued location: disengagement from fixation and shifting to the cued location. In the Gap condition, in contrast, removing the fixation cross before the onset of the cue allowed the disengagement process to be completed prior to the onset of the cue, leaving only the process of shifting attention to the cued location still to be completed. Subtracting response accuracy in the No-Gap condition (shift only) from accuracy in the Gap condition (disengage + shift), therefore, allows the disengagement component to be isolated and the speed of disengagement to be estimated. Mackeben and Nakayama (1993) found significantly more accurate performance in the Gap condition, when the fixation cross was removed 50–230 ms before the onset of the cue. This performance difference between the Gap and No-Gap conditions is known as the *gap effect*. Many variations of the gap paradigm have been employed to assess the disengagement of attention: some have measured RT or saccade latency instead of accuracy, others have employed a cue that was not predictive of the target location, and many have tested only a few locations rather than presenting a large array of distractors (e.g., Fisher & Weber, 1993; Gómez et al., 1998; Pratt & Nghiem, 2000; Tanaka & Shimojo, 2001). Importantly, the benefit gained from removing fixation—and from the consequent disengagement of attention—appears to be robust.

Since the goal of the present study was to assess whether the spatial extent of attention modulates the efficiency of disengagement, we combined a gap paradigm with an abrupt-onset central square that was either large or small to trigger the resizing of the attentional focus. Using abrupt-onset stimuli of different sizes, such as a small or large square, to trigger reflexive attentional resizing is common in the literature (e.g., Albonico et al., 2017; Burnett et al., 2013; Castiello & Umiltà, 1990; Galera et al., 2005; Gmeindl et al., 2020; Goodhew et al., 2017; Greenwood & Parasuraman, 1999, 2004; Maringelli & Umiltà, 1998; Turatto et al., 2000), and the attentional focus has been shown to reflexively expand or contract to match the size of the abrupt-onset object (e.g., Castiello & Umiltà, 1990; Maringelli & Umiltà, 1998; Turatto et al., 2000). Castiello and Umiltà (1990), for instance, found that RTs to detect a target presented inside of an abrupt-onset square increased by approximately 15 ms for every 1 degree increase in the size of the square, suggesting that the focus of attention increased in spatial extent as the size of the square increased. This resizing occurs even when resizing impairs task performance, which strongly suggests that it is a reflexive process (e.g., Turatto et al., 2000). Furthermore, studies displaying targets at a range of locations inside and outside of the large or small shape have confirmed that attention spreads over the entire region of the abrupt-onset shape, but not outside of it (e.g., Castiello & Umiltà, 1990, Experiment 2). Finally, attentional resizing occurs regardless of the location of the target inside the abrupt-onset shape and regardless of the predictability of the target’s location inside the shape (e.g., Castiello & Umiltà, 1990; Goodhew et al., 2017; Maringelli & Umiltà, 1998; Turatto et al., 2000).

**Fig. 1:**
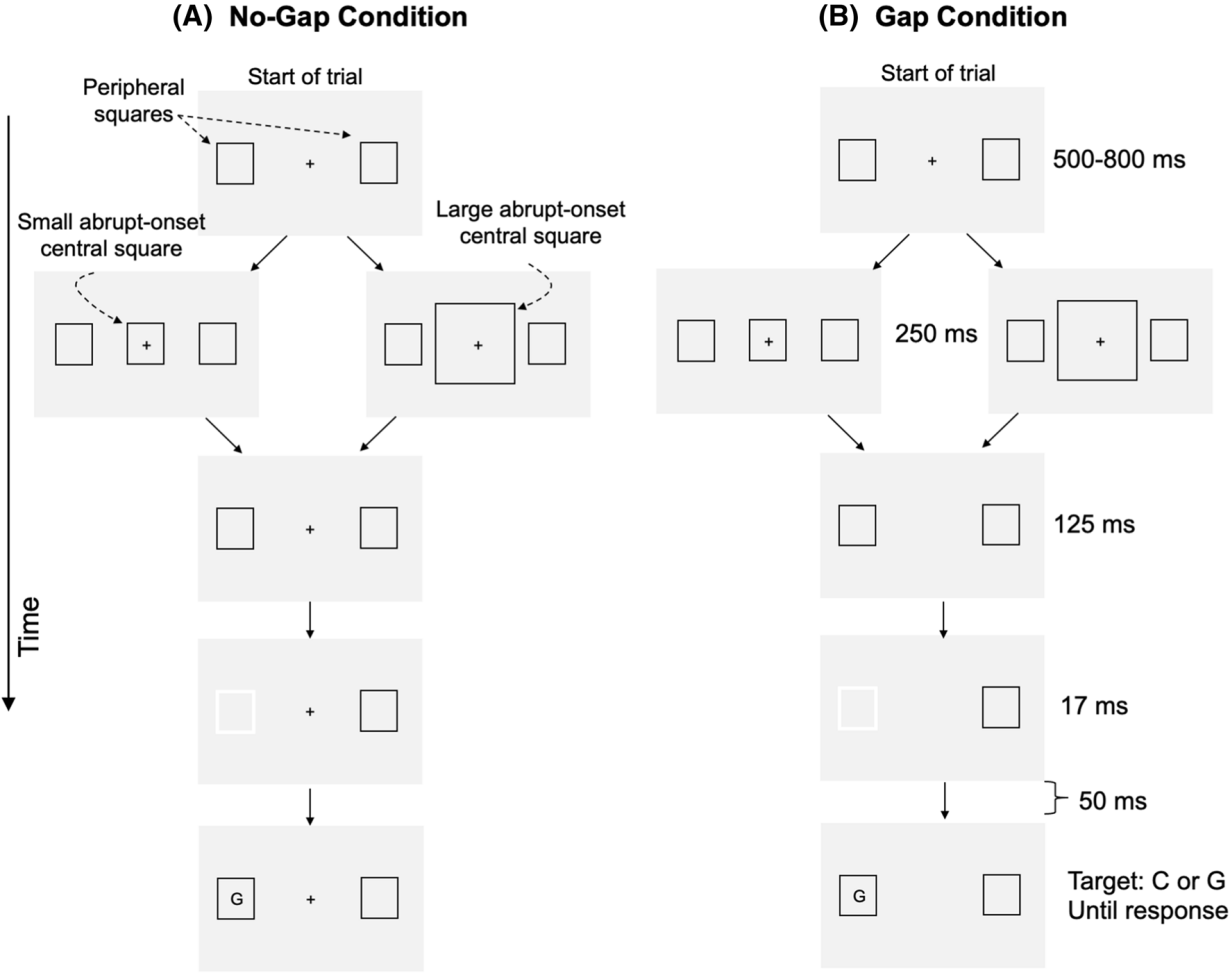
Schematic illustration of the paradigm in Experiment 1. The Gap and No-Gap conditions were randomly intermixed. Illustrated are trials in which the target appeared in the cued peripheral square; on 50% of the trials the target appeared in the uncued peripheral square

In the present Experiment 1, each trial began with a display containing a central fixation cross and two peripheral square outlines (referred to as *peripheral squares*), one to the left and one to the right of fixation (see Fig. 1). An abrupt-onset square (referred to as the *central square*) was then displayed, centered on fixation. The central square was either small or large, and its purpose was to trigger the reflexive resizing of attention. After a 250 ms delay, which is adequate to allow the resizing process to be completed (Maringelli & Umiltà, 1998), the fixation cross was removed in the Gap condition to trigger attentional disengagement. The fixation cross remained present in the No-Gap condition. One hundred twenty-five milliseconds later, one of the peripheral squares was cued by briefly changing colour from black to white to trigger reflexive orienting to its location. A letter target (C or G) then appeared unpredictably either inside the cued peripheral square (Valid condition) or inside the uncued peripheral square (Invalid condition), and participants made a speeded response to identify the letter.

The condition of interest is the Valid trials, in which the target appeared at the cued location. Invalid trials cannot be used to draw inferences about attentional disengagement, because those trials require an additional disengagement (from the cued peripheral square) and an additional attentional shift (from the cued to the uncued square). Invalid trials were included only to reduce the predictability of the target location, thereby discouraging participants from fixating the cued peripheral square. In Valid trials, the speed of attentional disengagement can be determined by subtracting RTs in the Gap condition from RTs in the No-Gap condition—the greater the difference, the larger the gap effect, and the less efficient the disengagement of attention. If the spatial extent of the attentional focus modulates the efficiency of disengagement, the magnitude of the gap effect will differ between the Small- and Large-central-square conditions. Specifically, if disengagement is less efficient when the attentional focus is small, the gap effect will be larger in the Small-square condition.

## Methods

### Participants

An a priori power analysis was conducted using G*Power (Faul et al. 2007) based on the effect size for the interaction between the magnitude of the gap effect and a secondary task requiring attention by Lester and Vecera (2018). The analysis indicated that a minimum sample of 52 participants is needed in order for an effect of that size to be detected with 90% probability with alpha set to 0.05.

Fifty-five undergraduate students participated in the experiment for course credit. Four participants were excluded from analysis: one reported a diagnosis of ADHD, one reported a diagnosis of OCD, two did not complete the experiment. This left a total of 51 participants for analysis. Forty-two participants self-identified as female, nine as male; two participants self-reported as left-handed; mean age = 22.8 years (SD = 6.35). All participants were naïve as to the purpose of the experiment, reported normal or corrected-to-normal vision, and provided written informed consent. This study and all further studies reported in this paper were approved by the Griffith University Human Research Ethics Committee and performed in accordance with the 1964 Declaration of Helsinki and its later amendments.

### Stimuli and procedures

All participants completed two tasks. The first was the *disengagement task*, as described above, which was designed to determine whether the spatial extent of attention changes the efficiency of disengagement. The second task was a *reflexive resizing task,* designed to confirm that the abrupt-onset central square triggered attentional resizing.

Participants completed the tasks in individual light- and sound-attenuated testing rooms. Stimuli were displayed on a 144-Hz BenQ XL2430T computer monitor powered by a Windows-based computer. Stimulus presentation was controlled by a custom Matlab script using Psyctoolbox 3 libraries (Brainard, 1997; Pelli, 1997; Kleiner et al., 2007). The room, computer, and software details were the same for all experiments reported in the present paper.

#### Disengagement task

Stimuli were black (2.3 cd/m^2^), displayed on a grey background (65 cd/m^2^). Each trial commenced with a fixation cross (0.2° × 0.2°) in the centre of the screen. Two square outlines (peripheral squares; 1° × 1°) were displayed, one 3° to the left and one 3° to the right of fixation (center-to-center; Fig. 1). The peripheral squares remained present throughout each trial. After a random delay of 500–800 ms, an abrupt-onset central square outline appeared, centered on fixation. On a randomly intermixed half of the trials, the abrupt-onset central square was small (1° × 1°); on the remaining trials it was large (4° × 4°). The abrupt-onset central square remained visible for 249.8 ms before being removed. After a delay of 124.9 ms, one of the peripheral squares (randomly either the left or the right square) was cued by changing from black to white (129 cd/m^2^) for 17 ms. After a 48.6 ms delay, a letter (C or G; 0.5°) appeared randomly but with equal probability either centered inside the cued peripheral square or inside the uncued square. Participants identified the letter by pressing a key labelled “C” or “G” as quickly as possible. If responses were too slow (> 1200 ms) or if participants made too many errors in identifying the target (after 8 errors), a 1-s warning message was displayed on the screen.

In the No-Gap condition the fixation cross remained present and the trial was as described above. The Gap condition was identical to the No-Gap condition except that the fixation cross was removed at the same time as the central square. The Gap and No-Gap conditions were randomly intermixed. After each trial there was a blank interval of 1000 ms before the next trial commenced. Participants completed ten practice trials to familiarize themselves with the task, followed by 288 experimental trials.

#### Reflexive resizing task

Each trial commenced with a fixation cross (0.2° × 0.2°) displayed in the centre of the screen. After a random 600–1800 ms delay, the outline of a square appeared on the screen, centred on the fixation cross. On a random half of the trials the square was small (1° × 1°); on the remaining trials it was large (4° × 4°). 254 ms after the onset of the square, a letter (C or G, with equal probability) replaced the fixation cross, and participants pressed a key labelled “C” or “G” as quickly as possible to identify the target. Participants completed 160 trials. A warning was displayed on the screen if responses were too slow or if the participant made too many errors in identifying the target.

## Results

### Reflexive resizing task

**Fig. 2:**
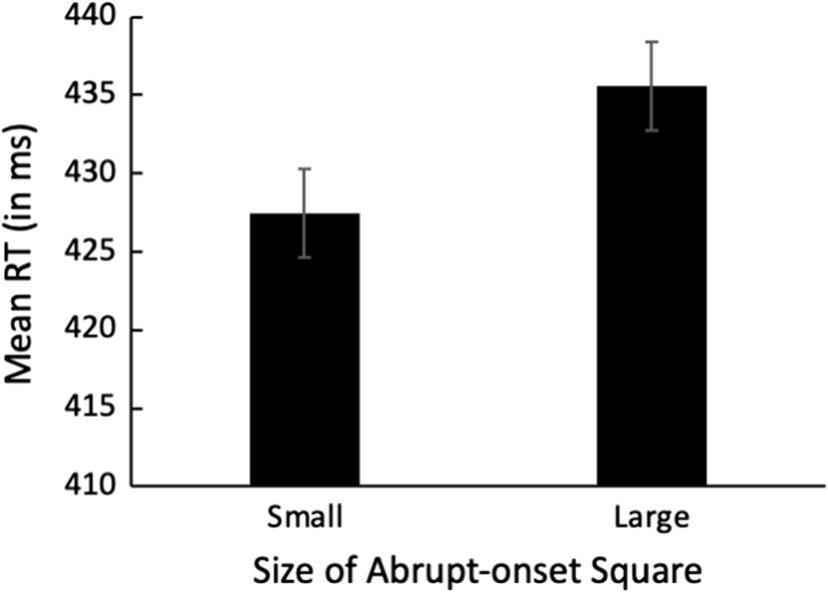
Mean RT in milliseconds as a function of the size of the abrupt-onset square (small or large). Error bars indicate ± one standard error of the mean

Trials with RTs ± 2 SD from the mean were removed as outliers prior to analysis. Mean RT as a function of the size of the abrupt-onset square is illustrated in Fig. 2. Mean target identification accuracy was 94.5% (SD = 3.23). A *t* test revealed that RTs were significantly faster in the Small-square condition than in the Large-Square condition, *t*(50) = 3.87, *p* < 0.001, *d* = 0.543, consistent with the expectation that the abrupt-onset squares triggered the reflexive resizing of the attentional focus.

### Disengagement task

#### Accuracy results

Reaction time is the primary measure of interest in this study. However, before proceeding to consider the RT data in greater detail, we first consider the accuracy data to determine whether there are speed–accuracy trade-offs that might affect our interpretation.

In the Valid condition, mean target identification accuracy was 95.53% (SD = 4.72) in the Small-Square Gap condition, 94.90% (SD = 4.52) in the Small-Square No-Gap condition, 96.26% (SD = 4.56) in the Large-Square Gap condition, and 93.63% (SD = 4.96) in the Large-Square No-Gap condition. A 2 (Central Square Size: Small, Large) × 2 (Gap Presence: No Gap, Gap) repeated-measures analysis of variance (ANOVA) yielded a significant main effect of Gap Presence, *F*(1,50) = 10.79, *p* = 0.002, *ƞ*_p_^2^ = 0.177. The main effect of Central Square Size was not significant, *F* < 1. The interaction between Gap Presence and Central Square Size was significant, *F*(1,50) = 6.727, *p* = 0.012, *ƞ*_p_^2^ = 0.119. In considering whether these results are evidence of a speed–accuracy trade-off, we note that the condition in which RTs are slowest (No-Gap, Small-Square condition) is associated with lower levels of accuracy; the condition in which RTs are fastest (Gap, Small-Square condition) is associated with higher levels of accuracy. Thus, there is no indication of a speed–accuracy trade off in the Valid condition of Experiment 1.

In the Invalid condition, mean target identification accuracy was 93.72% (SD = 3.98) in the Small-Square Gap condition, 94.15% (SD = 5.13) in the Small-Square No-Gap condition, 94.91% (SD = 3.86) in the Large-Square Gap condition, and 95.24% (SD = 4.58) in the Large-Square No-Gap condition. A 2 (Central Square Size: Small, Large) × 2 (Gap Presence: No Gap, Gap) repeated-measures ANOVA yielded a significant main effect of Central Square Size, *F*(1,50) = 5.67, *p* = 0.021, *ƞ*_p_^2^ = 0.102, indicating that accuracy was, on average, lower in the Small-Square condition. The main effect of Gap Presence was not significant, *F* < 1. The absence of a significant interaction, F < 1, indicates there is no speed–accuracy trade-off in the Invalid condition.

#### Reaction time results

**Fig. 3:**
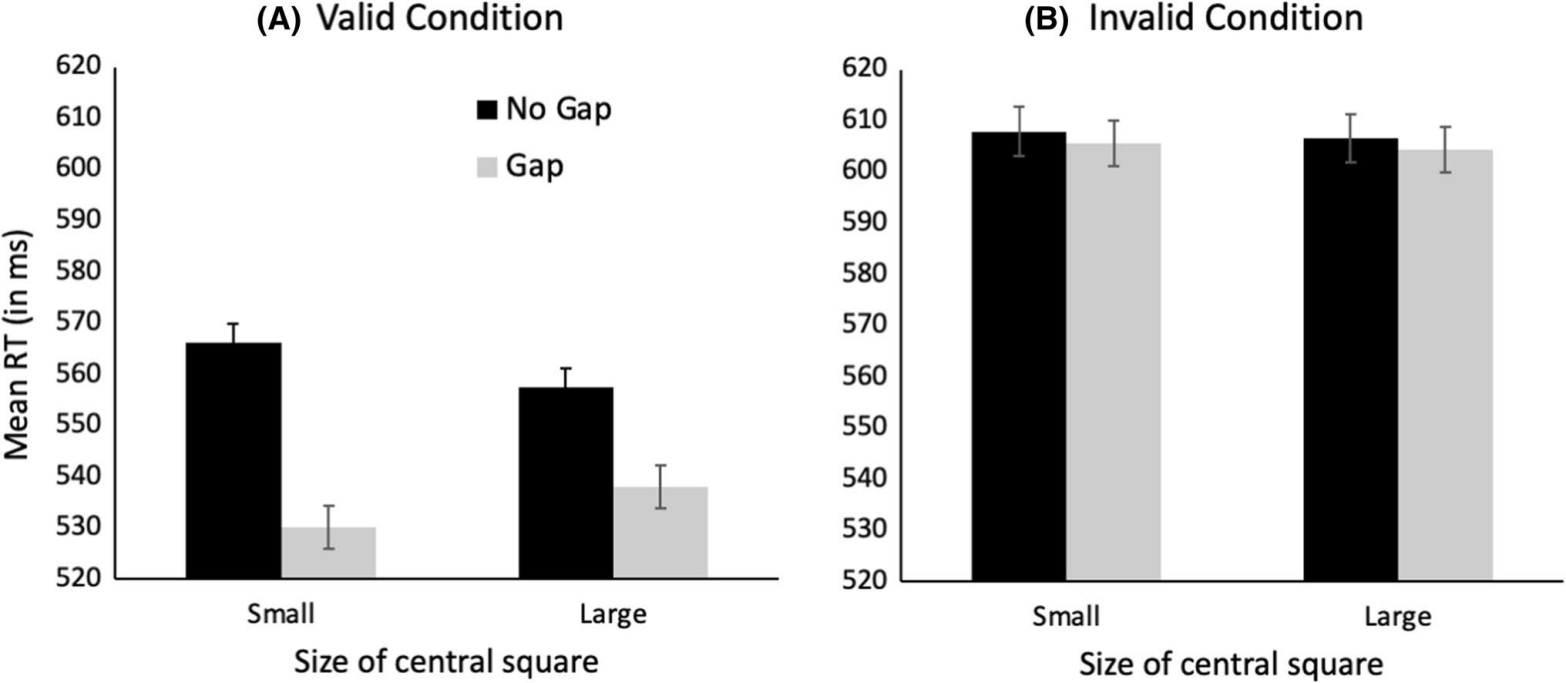
Mean RT in milliseconds in the disengagement task as a function of central square size and gap presence in the Valid condition (**A**) and the Invalid condition (**B**). Error bars indicate ± one standard error of the mean

Trials with RTs ± 2 SD from the mean were excluded from analysis. Only trials on which the target was correctly identified were included for analysis. Mean RT as a function of the size of the abrupt-onset central square and the presence of a gap is illustrated in Fig. 3.

The data in Fig. 3 were analyzed in an overall 2 (Validity: Valid, Invalid) × 2 (Gap Presence: Gap, No Gap) × 2 (Size of central square: Small, Large) repeated-measures ANOVA. The analysis revealed significant main effects of Validity, *F*(1,50) = 250.8, *p* < 0.001, *ƞ*_p_^2^ = 0.805, and Gap Presence, *F*(1,50) = 69.40, *p* < 0.001, *ƞ*_p_^2^ = 0.581. There were also significant interactions between Square Size and Gap Presence, *F*(1,50) = 8.11, *p* = 0.006, *ƞ*_p_^2^ = 0.140, and Gap Presence and Validity, *F*(1,50) = 36.91, *p* < 0.001, *ƞ*_p_^2^ = 0.425. Most importantly, the three-way interaction among Square Size, Gap Presence, and Validity was significant, *F*(1,50) = 6.40, *p* = 0.015, *ƞ*_p_^2^ = 0.113. No other main effects or interactions were significant.

Our next step was to analyze the results of the Valid and Invalid conditions separately. As outlined in the Introduction, our interest lies in the Valid condition, since this is the only condition that allows the disengagement process to be isolated. It was expected that all RTs in the Invalid condition would be comparable to one another. This was confirmed by 2 (Size of central square: Small, Large) × 2 (Gap Presence: Gap, No Gap) repeated-measures ANOVA, which yielded no significant main effects or interactions, all *F*s < 1. The data in the Valid condition were analyzed in a 2 (Size of central square: Small, Large) × 2 (Gap Presence: Gap, No Gap) repeated-measures ANOVA. The analysis revealed a significant main effect of Gap Presence, *F*(1,50) = 91.44, *p* < 0.001, *ƞ*_p_^2^ = 0.646. The main effect of Square Size was not significant, *F* < 1. Critically, the interaction between Square Size and Gap Presence was significant, *F*(1,50) = 15.32, *p* = 0.001, *ƞ*_p_^2^ = 0.235. This significant interaction indicates that the RT benefit arising from the presence of a gap is greater in the Small-Square condition (35.9 ms) than in the Large-Square condition (19.4 ms), suggesting that attentional disengagement is slower when the spatial extent of the attentional focus is small, *t*(50) = 3.92, *p* < 0.001, *d* = 0.548.

Although the significant interaction is consistent with the interpretation that disengagement is slower when the attentional focus is small, there is an unexpected aspect to the results illustrated in Fig. 3. Specifically, RTs differ significantly between the Small- and the Large-Square conditions in both the No-Gap condition, *t*(50) = 2.71, *p* = 0.009, *d* = 0.380, and the Gap condition, *t*(50) = 2.35, *p* = 0.023, *d* = 0.329. While it is expected that a significant RT difference would occur in the No-Gap condition due to differences in the amount of time required to disengage a small versus a large attentional focus, the significantly faster RTs in the Gap condition when the central square is small is not expected. The removal of the central fixation cross in the Gap condition should trigger the disengagement of attention, thus facilitating a rapid attention shift. This disengagement is expected to occur regardless of the size of the central square, and thus RTs should be equal regardless of the size of the central square. The fact that RTs were significantly faster in the Small-Square condition, therefore, suggests there is an additional factor at play.

One likely factor is changes to the rate of visual processing when the attentional focus is narrow or broad. As outlined earlier, there is a trade-off between the spatial extent of the attentional focus and the speed and resolution of visual processing within the attended area (e.g., Eriksen & Yeh, 1985; Eriksen & St. James, 1986), such that stimuli encompassed by a narrow attentional focus are processed more rapidly and accurately than stimuli encompassed by a broader attentional focus (Castiello & Umiltà, 1990). There are many studies showing that the size of the attentional focus influences the speed at which the onset of a stimulus can be detected (e.g., Castiello & Umiltà, 1990; Maringelli & Umiltà, 1998; Turatto et al., 2000), and it seems likely that the size of the focus would also influence the speed at which a stimulus *offset* can be detected. If so, fixation offset in the Gap condition would be detected earlier in the Small-Square than in the Large-Square condition, triggering earlier disengagement with the presentation of the small central square and reducing RTs in that condition. Experiment 2 was designed to test whether the offset of a stimulus can be detected more rapidly when the attentional focus is narrow.

## Experiment 2

### Participants

An a priori power analysis was conducted using G*Power (Faul et al. 2007) based on the effect size in the focusing task of Experiment 1. The analysis indicated that a minimum sample of 31 participants would be needed for an effect of that size to be detected with 90% probability with alpha set to 0.05.

Twenty-nine undergraduate students participated in the experiment for course credit. Twenty-three self-identified as female, six as male; 28 were right-handed, one was left-handed; mean age was 23.0 years (SD = 5.81) All participants were naïve as to the purpose of the experiment, reported normal or corrected-to-normal vision, and provided written informed consent.

### Stimuli and procedures

Each trial commenced with a fixation cross (0.2° × 0.2°) displayed in the centre of the screen. After a random 600–1800 ms delay, the outline of a square appeared on the screen, centred on the fixation cross. On a random half of the trials the square was small (1° × 1°); on the remaining trials it was large (4° × 4°). 254 ms after the onset of the square, the central fixation cross disappeared, and the task of the participants was to press the ‘b’ key as quickly as possible when they detected the offset of the fixation cross. Participants completed 192 trials.

### Results and discussion

**Fig. 4:**
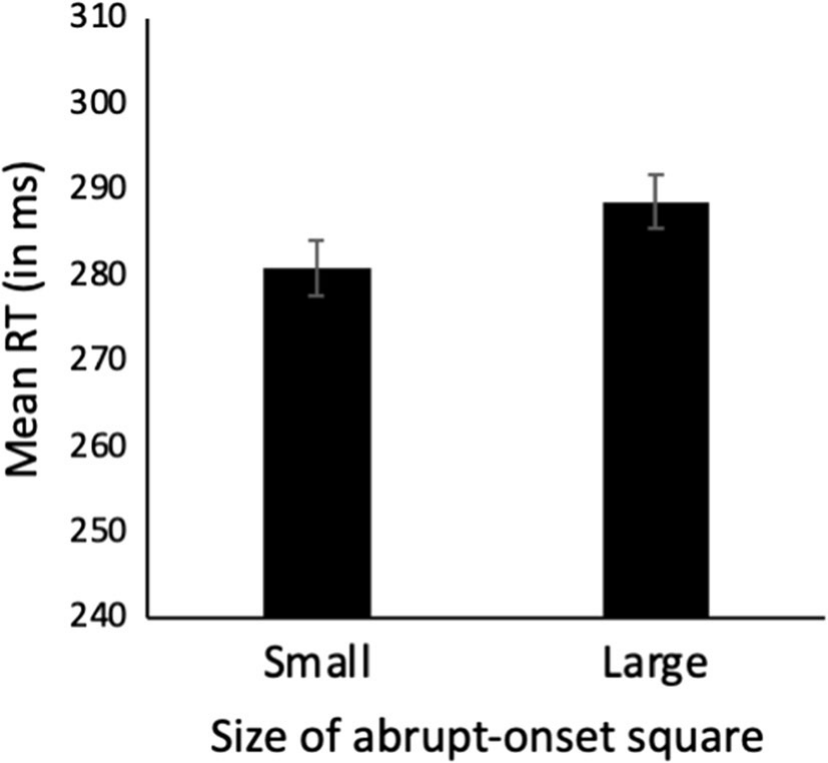
Mean RT in milliseconds to detect the offset of central fixation, illustrated as a function of the size of the abrupt-onset square (small or large). Error bars indicate ± one standard error of the mean

As in Experiment 1, any trials with RTs ± 2 SD from the mean were removed as outliers prior to analysis. Mean RT as a function of the size of the abrupt-onset square is illustrated in Fig. 4. A *t* test indicated that participants were significantly faster at detecting the offset of the fixation cross in the Small-Square condition than in the Large-Square condition, *t*(28) = 2.74, *p* < 0.01, *d* = 0.508. Specifically, participants were 7.8 ms faster at detecting the fixation offset in the Small-Square condition. This is notably comparable to the 7.9 ms decrease in RTs in the Small-Square Gap condition relative to the Large-Square Gap condition in Experiment 1 (Fig. 3, panel A, gray bars), suggesting that the differences in the speed of detecting fixation offset as a function of the size of the attentional focus likely underlie the unexpected RT difference in the Gap condition in Experiment 1.

## Experiment 3

The results of Experiments 1 and 2 strongly suggest that the spatial extent of the attentional focus modulates the efficiency of attentional disengagement. In Experiment 3 we employed an adapted version of the paradigm used in Experiment 1—in this modified paradigm there were no peripheral squares and no luminance change to trigger a reflexive shift of attention to the peripheral square’s location. Instead, observers were simply presented with a central fixation cross, followed by the onset of a small or large abrupt-onset central square. As in Experiment 1, central fixation then either remained present (No-Gap condition) or was removed (Gap condition). This was followed by the onset of the target displayed randomly and unpredictably either to the left or the right of the central square.

Experiment 3 with its modified paradigm served three purposes. First, it provided a replication of the results in Experiment 1. Second, it is known that when a shape to which attention can be anchored is displayed throughout a trial, it can change how attention is distributed (e.g., Jefferies & Di Lollo, 2015, 2017). Removing the peripheral boxes avoids this potential issue. Finally, it is possible that participants in Experiment 1 fixated the location of one of the peripheral squares, particularly as one square was cued by a change in luminance, which might have unintentionally served as an exogenous trigger for a saccade. This exogenous trigger is eliminated by the removal of the peripheral squares and thus also the removal of the luminance change.

### Participants

An a priori power analysis was conducted using G*Power (Faul et al. 2007) based on the effect size (*ƞ*_p_^2^ = 0.235) for the interaction between Gap Presence and Central Square Size in the Valid condition in Experiment 1. The analysis indicated that a minimum sample of 22 participants would be needed for an effect of that size to be detected with 95% probability with alpha set to 0.05.

Thirty-eight undergraduate students participated in the experiment for course credit. Three participants were removed from the analysis: one reported a diagnosis of OCD, one for excessively long RTs that averaged 3 standard deviations above the average, and one due to disruptions during the experiment. This left a total of 33 participants for analysis. Nineteen self-identified as female, 12 as male, 2 as non-binary, and 1 declined to answer. One participant self-reported as left-handed, 1 as ambidextrous, and 31 as right-handed; mean age = 20.18 years (SD = 3.16). All participants were naïve as to the purpose of the experiment, reported normal or corrected-to-normal vision, and provided written informed consent.

### Stimuli and procedures

**Fig. 5:**
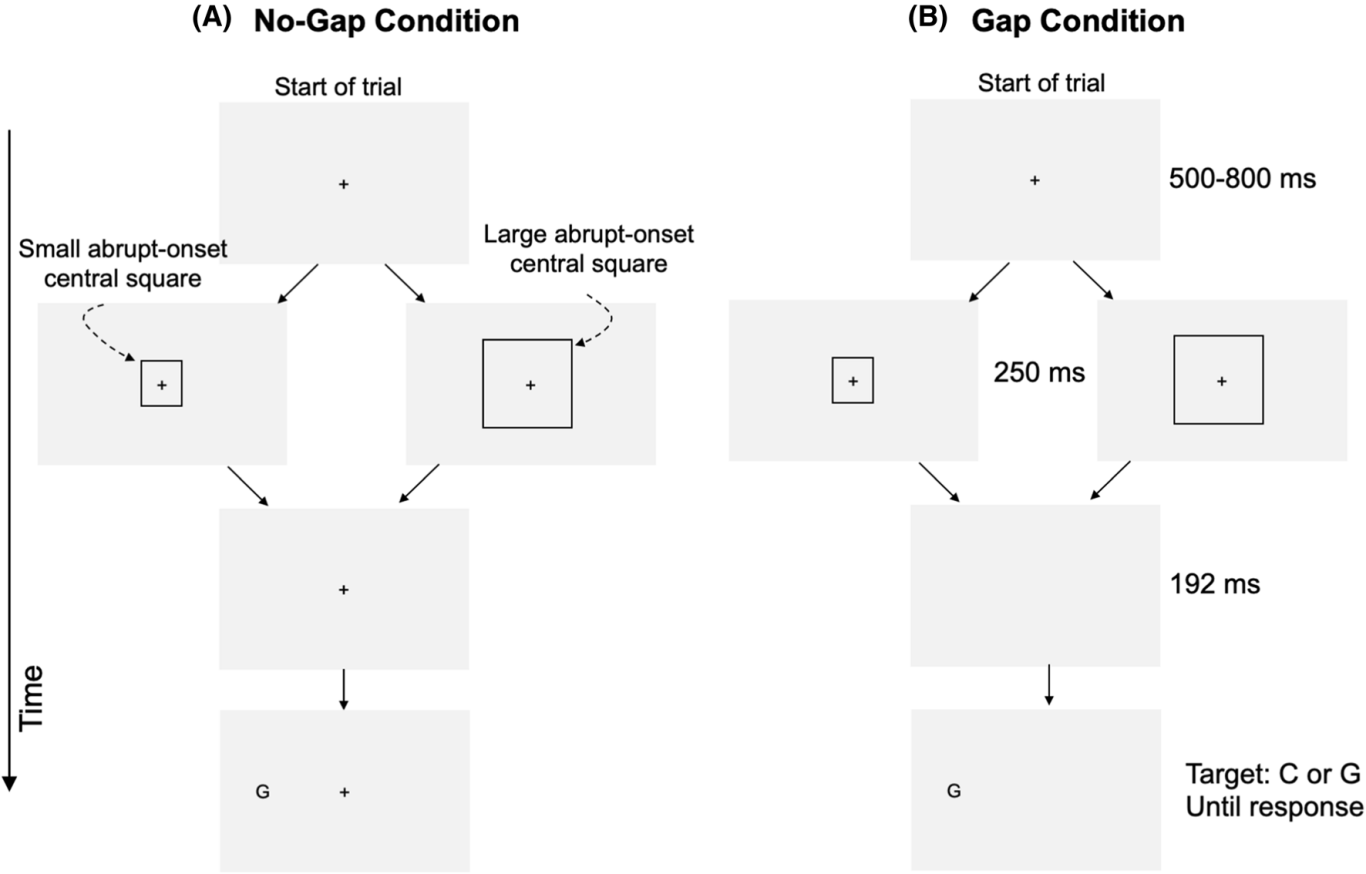
Schematic illustration of the No-Gap and Gap conditions of Experiment 3

The stimuli and procedures in Experiment 3 were the same as in Experiment 1 with the following changes. There were no peripheral squares in the display, and since there were no peripheral squares, there was also no colour change of one of the squares (see Fig. 5).

The sequence of events was as follows. After a delay of 500–800 ms, an abrupt-onset central small or large square outline appeared, centered on fixation. The abrupt-onset central square remained visible for 249.8 ms before being removed. When the central square was removed, the fixation cross either remained present (No-Gap condition) or it was removed along with the central square (Gap condition). After a delay of 192 ms, a letter (C or G) appeared randomly but with equal probability either to the left or to the right of fixation, at the same location as in Experiment 1.

### Results and discussion

**Fig. 6:**
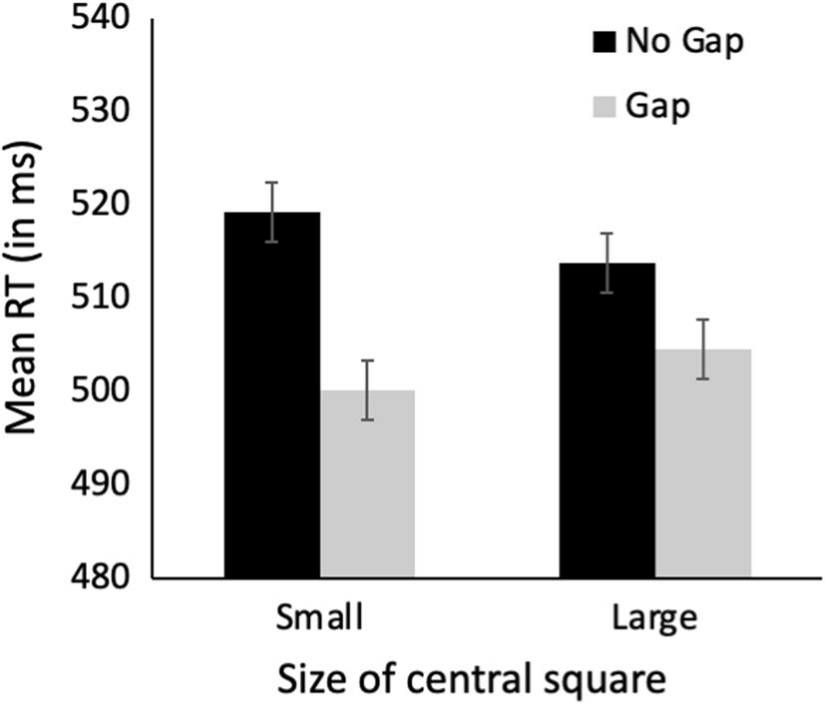
Mean RT in milliseconds as a function of central square size and gap presence. Error bars indicate ± one standard error of the mean

Trials with RTs ± 2 SD from the mean were excluded from analysis. Only trials on which the target was correctly identified were included for analysis. Mean RT as a function of the size of the abrupt-onset central square and the presence of a gap is illustrated in Fig. 6.

### Accuracy results

As in Experiment 1**,** reaction time is the primary measure of interest. However, before proceeding to consider RT data, we first consider the accuracy data to determine whether there are any speed–accuracy trade-offs. Mean target identification accuracy was 94.76% (SD = 4.50) in the Small-Square Gap condition, 93.8% (SD = 3.49) in the Small-Square No-Gap condition, 94.63% (SD = 3.50) in the Large-Square Gap condition, and 95.22%(SD = 3.64) in the Large-Square No-Gap condition. A 2 (Central Square Size: Small, Large) X 2 (Gap Presence: No Gap, Gap) repeated-measures ANOVA yielded no significant effects. The main effect of Central Square Size was not significant, *F*(1,33) = 2.912, *p* = 0.097, nor was the main effect of Gap Presence, *F* < 1. The interaction effect was marginally significant, *F*(1,33) = 3.76, *p* = 0.061. Since accuracy is higher and RTs are faster in the Small-Square Gap condition, while accuracy is lower and RTs are slower in the Small-Square No-Gap condition, there is no evidence of a speed–accuracy trade-off. Similarly, accuracy is lower and RTs are faster in the Large-Square Gap condition, while accuracy is higher and RTs are slower in the Large-Square No-Gap condition, indicating no speed–accuracy trade-off.

### Reaction time results

The data illustrated in Fig. 6 were analyzed in a 2 (Gap Presence: Gap, No Gap) × 2 (Size of central square: Small, Large) repeated-measures ANOVA. The analysis revealed a significant main effect of Gap Presence, *F*(1,33) = 34.20, *p* < 0.001, *ƞ*_p_^2^ = 0.805, but the main effect of Square Size was not significant, *F* < 1. Critically, the interaction between Gap Presence and Square Size was significant, *F*(1,33) = 12.65, *p* = 0.001, *ƞ*_p_^2^ = 0.277. This significant interaction indicates that the RT benefit arising from the presence of a gap (i.e., No-Gap RT minus Gap RT) is greater in the Small-Square condition (19.1 ms) than in the Large-Square condition (9.2 ms), suggesting that attentional disengagement is slower when the spatial extent of the attentional focus is small, *t*(33) = 3.55, *p* < 0.001, *d* = 0.608. Follow-up *t* tests showed that, as in Experiment 1, RTs differ significantly between both the Small- and the Large-Square conditions in both the No-Gap, (*t*(3) = 2.18, *p* = 0.037, *d* = 0.373), and the Gap conditions, (*t*(33) = 2.05, *p* = 0.049, *d* = 0.351).

The results of Experiment 3 closely replicate those of Experiment 1. The significant interaction indicates that the magnitude of the gap effect is greater when the central square is small than when the central square is large, an effect that arises primarily from the fact that RTs were significantly slower in the No-Gap condition when the central square is small. This pattern of results indicates that disengagement is delayed when attention is narrowly focused, confirming the results of Experiment 1. In addition, since Experiment 3 did not include the peripheral boxes that were part of the display in Experiment 1, and since there was no brightening of one of the boxes, which might have served as an exogenous trigger for a saccade, the present findings also suggest that the results do not stem from observers fixating the peripheral boxes. As a further consideration, there is also no strategic reason for observers to fixate one of the peripheral locations. The target appears unpredictably to either the left or right side, and thus its location cannot be predicted on any given trial. Fixating one peripheral location in advance of the target appearing would mean attending to the wrong location on 50% of the trials, thus hindering performance on the task. Given the above considerations, it seems unlikely that observers made anticipatory eye movements.

## Experiment 4

In both Experiments 1 and 3, the duration of the central square and the temporal delay between the offset of the central square and the onset of the target were fixed. In Experiment 3, for example, the central square was always displayed for 250 ms and the target invariably appeared 192 ms after the removal of the central square. In consequence, participants could accurately estimate when (although not where) the target would appear, and could potentially use that knowledge to optimize their performance on the task by increasing their readiness to disengage or orient attention. To address this, Experiment 4 eliminated the temporal predictability of the target by varying both the exposure duration of the central square (50 ms or 288 ms) and the duration of the temporal delay between the offset of the central square and the onset of the target (144 ms or 288 ms). Furthermore, as in Experiments 1 and 3, the fixation cross offset at the same time as the central square in the Gap condition; consequently, the temporal delay between the offset of fixation and the onset of the target was also either 144 ms or 288 ms. These temporal variations mean that the onset time of the target cannot be predicted.

Although the primary purpose of Experiment 4 was to reduce the temporal predictability of the paradigm, we also used Experiment 4 as an opportunity to further test whether varying the spatial extent of the attentional focus affects the efficiency of attentional disengagement using a very short as well as a long exposure duration for the central square. If the exposure duration of the central square is long enough to allow adequate time for the focusing process to be completed, attention will be broad in the Large-Square condition and narrow in the Small-Square condition at the time that it is disengaged. If, on the other hand, the exposure duration is too short to allow the focusing process to be completed, the size of the attentional focus will not differ between the Small- and Large-Square conditions and there will be no effect of central square size.

In deciding on the short duration for the central square, we drew on the findings of Castiello and Umiltà (1990) who found that when the target appeared just 50 ms after a small or large abrupt-onset square, RTs did not vary as a function of square size. They attributed this to the fact that 50 ms was insufficient time for the focus of attention to resize. Maringelli and Umiltà (1998) subsequently found evidence of attentional resizing at 100 ms or more. Based on this, we decided to use a short exposure duration of 50 ms and a longer exposure duration of 288 ms in Experiment 4.

We have two specific predictions for Experiment 4. First, when the exposure duration of the central square is just 50 ms, there will be insufficient time to resize the attentional focus, and there should be no effect of central square size on the efficiency of attentional disengagement (i.e., there should be no effect of central square size on the magnitude of the gap effect). Second, if the temporal predictability of the target in Experiments 1 and 3 is not the cause of the observed results, then when the exposure duration of the central square is sufficiently long to allow attention to be resized (288 ms), we should see an effect of central square size on the speed of attentional disengagement (the magnitude of the gap effect), replicating the results of Experiment 3.

Although the discussion above focuses on the exposure duration of the central square, in Experiment 4 we also manipulated the temporal delay between the offset of the central square and the onset of the target. Unlike the exposure duration of the central square, the duration of the temporal gap between the offset of the central square and the onset of the target will not affect the resizing of attention. Thus, we expect to see an effect of the breadth of attention on the speed of disengagement (i.e., the size of the central square should interact with the magnitude of the gap effect), as in Experiment 3.

## Methods

### Participants

An a priori power analysis was conducted using G*Power (Faul et al. 2007). We initially selected the effect size for the interaction between Gap Presence and Central Square Size in the Valid condition in Experiment 3 (*ƞ*_p_^2^ = 0.277). However, since we wished to have adequate power to pick up potentially very small effects in the short timing conditions, we opted to use an effect size of half that obtained in Experiment 3 (i.e., *ƞ*_p_^2^ = 0.133). The analysis indicated that a minimum sample of 36 participants would be needed for an effect of that size to be detected with 90% probability with alpha set to 0.05.

Forty-two undergraduate students (mean age = 22.48 years, SD = 7.07; 31 participants self-identified as female, 11 as male) participated in the experiment for course credit. One participant was removed from the analysis due to reporting an in-progress diagnosis of OCD, leaving a total of 41 participants for analysis. All were naïve as to the purpose of the experiment, reported normal or corrected-to-normal vision, and provided written informed consent.

### Stimuli and procedures

The stimuli and procedures were identical to those in Experiment 3 except that the temporal predictability of the target was removed. In Experiment 4, the exposure duration of the central square was short (50 ms) on a randomly intermixed 50% of the trials and long (288 ms) on the remaining trials. The temporal delay between the offset of the central square and the onset of the target was also made unpredictable, being short (144 ms) on a randomly intermixed 50% of the trials and long (288 ms) on the remaining trials. Given these timing manipulations, the temporal onset of the target could not be reliably predicted.

## Results and discussion

### Accuracy of responding

As in Experiments 1 and 3, reaction time is the primary measure of interest; however, we first consider the accuracy data. Mean target identification accuracy 95.9% (SD = 2.9). This is comparable to the accuracy of target identification in Experiments 1 (94.5%) and 3 (94.6%), indicating that participants were able to accurately identify the target regardless of whether it was temporally predictable (as in Experiments 1 and 3) or temporally unpredictable (as in the current experiment). To examine the data for any speed–accuracy trade-offs, the accuracy for each condition is considered below.

#### Central square long, temporal gap duration short

Mean target identification accuracy was 96.0% in the Small-Square Gap condition, 96.45% in the Small-Square No-Gap condition, 94.91% in the Large-Square Gap condition, and 95.77% in the Large-Square No-Gap condition. A 2 (Central Square Size: Small, Large) × 2 (Gap Presence: No Gap, Gap) repeated-measures ANOVA yielded no significant main effects (Central Square Size, *F*(1,40) = 1.47, *p* = 0.231, Gap Presence, *F* < 1) or significant interaction, *F* < 1. This indicates that accuracy of responding was comparable in all conditions, with no evidence of a speed–accuracy trade-off.

#### Central square long, temporal gap duration long

Mean target identification accuracy was 95.38% in the Small-Square Gap condition, 96.35% in the Small-Square No-Gap condition, 95.24% in the Large-Square Gap condition, and 97.62% in the Large-Square No-Gap condition. A 2 (Central Square Size: Small, Large) × 2 (Gap Presence: No Gap, Gap) repeated-measures ANOVA yielded a marginal main effect of Gap Presence, *F*(1,40) = 3.85, p = 0.057. The main effect of Central Square Size was not significant, *F*(1,40) = 1.61, *p* = 0.212, nor was the interaction effect, *F* < 1. Since accuracy does not vary as a function of Central Square Size, and since Gap Presence does not interact with Central Square Size, there is no indication of a speed–accuracy trade-off.

#### Central square short, temporal gap duration short

Mean target identification accuracy was 95.53% in the Small-Square Gap condition, 96.02% in the Small-Square No-Gap condition, 94.52% in the Large-Square Gap condition, and 96.36% in the Large-Square No-Gap condition. A 2 (Central Square Size: Small, Large) × 2 (Gap Presence: No Gap, Gap) repeated-measures ANOVA yielded no significant main effect of Central Square Size, *F*(1,40) = 2.21, *p* = 0.145, was Gap Presence, *F* < 1, or the interaction between them, *F* < 1. This indicates that accuracy of responding was comparable in all conditions, with no evidence of a speed–accuracy trade-off.

#### Central square short, temporal gap duration long

Mean target identification accuracy was 96.28% in the Small-Square Gap condition, 96.92% in the Small-Square No-Gap condition, 95.37% in the Large-Square Gap condition, and 95.96% in the Large-Square No-Gap condition. A 2 (Central Square Size: Small, Large) × 2 (Gap Presence: No Gap, Gap) repeated-measures ANOVA yielded no significant main effect of Central Square Size, *F*(1,40) = 1.41, *p* = 0.242, Gap Presence, *F* < 1, or the interaction between them, *F* < 1. This indicates that accuracy of responding was comparable in all conditions, with no evidence of a speed–accuracy trade-off.

### Reaction time results

**Fig. 7:**
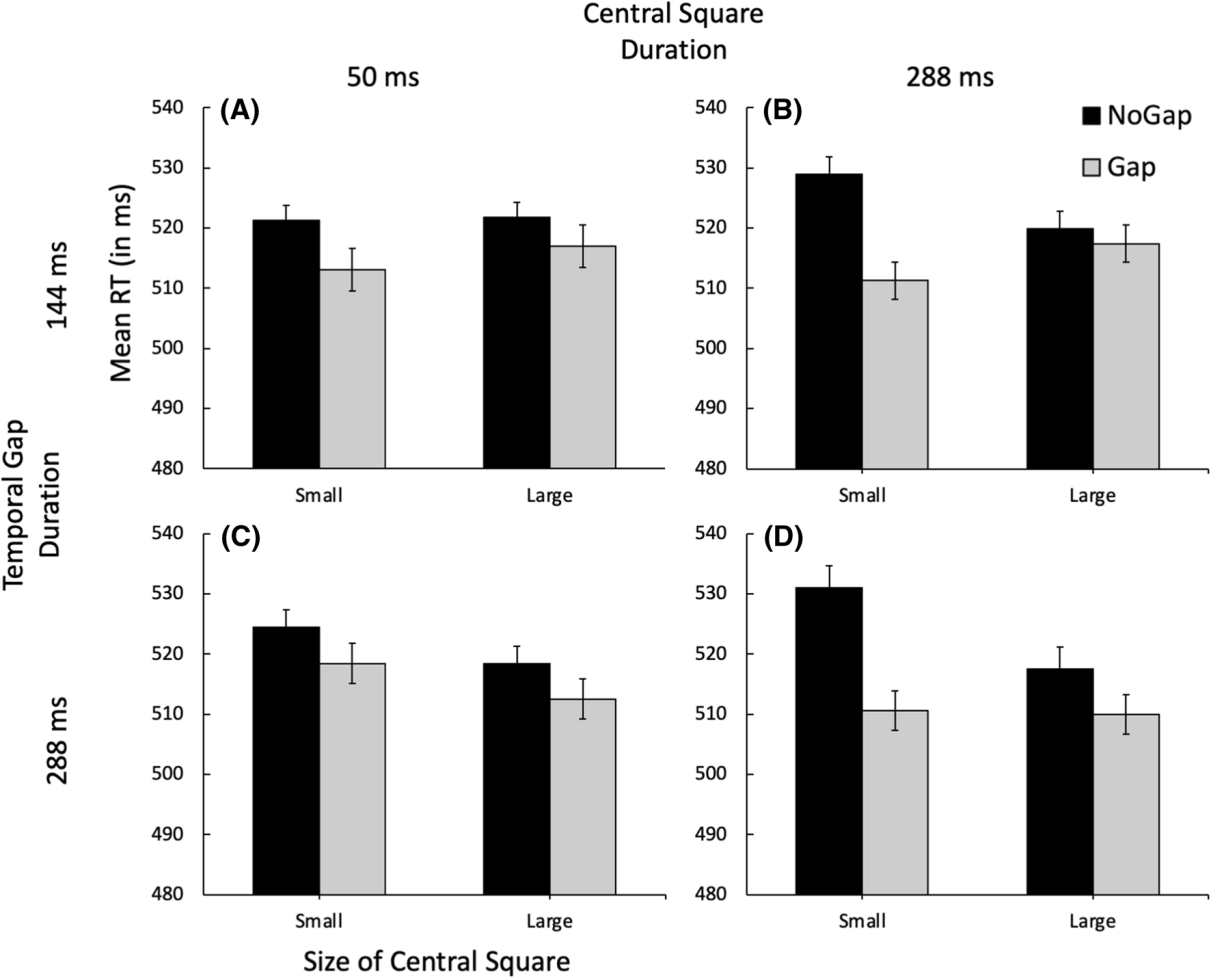
Mean RT in milliseconds as a function of central square size, gap presence, central square duration, and temporal gap duration. Error bars indicate ± one standard error of the mean

As in the previous experiments, trials with RTs ± 2 SD from the mean were excluded from analysis. Only trials on which the target was correctly identified were included for analysis. Mean RT as a function of the size of the abrupt-onset central square and the presence of a gap is illustrated separately in Fig. 7 for each combination of the duration of the central square (Central Square Duration: 50 ms or 288 ms) and the duration of the temporal gap between the offset of the central square and the onset of the target (Temporal Gap Duration: 144 ms or 288 ms).

The data illustrated in Fig. 7 were analyzed in four separate 2 (Gap Presence: Gap, No Gap) × 2 (Size of central square: Small, Large) repeated-measures ANOVA, one for each combination of Central Square Duration and Temporal Gap Duration (i.e., a separate analysis for each graph illustrated in Fig. 7).

#### Central square long, temporal gap duration short (Fig. 7, panel B)

The main effect of Gap Presence was significant, *F*(1,40) = 8.131, *p* = 0.007, *ƞ*_p_^2^ = 0.169. Critically, the Gap Presence × Square Size interaction was significant, *F*(1,40) = 4.941, *p* = 0.032, *ƞ*_p_^2^ = 0.110, indicating that the magnitude of the Gap Effect varied as a function of the size of the central square.

#### Central square long, temporal gap duration long (Fig. 7, panel D)

The main effect of Gap Presence was significant, *F*(1,40) = 15.47, *p* < 0.001, *ƞ*_p_^2^ = 0.279. Critically, the Gap Presence × Square Size interaction was significant, *F*(1,40) = 4.15, *p* = 0.048, *ƞ*_p_^2^ = 0.094, indicating that the magnitude of the Gap Effect varied as a function of the size of the central square.

#### Central square short, temporal gap duration short (Fig. 7, panel A)

The main effect of Square Size was not significant, *F* < 1, nor was the main effect of Gap Presence, *F*(1,40) = 2.904, *p* = 0.096, *ƞ*_p_^2^ = 0.068. Importantly, the Square Size × Gap Presence interaction was not significant, *F* < 1.

#### Central square short, temporal gap duration long (Fig. 7, panel C)

The main effect of Square Size was not significant, *F*(1,40) = 3.25, *p* = 0.079, *ƞ*_p_^2^ = 0.075, nor was the main effect of Gap Presence, *F*(1,40) = 3.15, *p* = 0.083, *ƞ*_p_^2^ = 0.073. Importantly, the Square Size × Gap Presence interaction was not significant *F* < 1.

As expected, only in those conditions which allowed sufficient time for the attentional focus to resize (i.e., when the Central Square Duration was long; Fig. 7B, D) was there an effect of central square size on the magnitude of the gap effect. In those conditions, the magnitude of the gap effect is greater when the central square is small than when it is large, closely replicating the results of Experiment 3. The results of Experiment 4 also show that even when the timing of the target is unpredictable, the disengagement of attention is less efficient when the focus of attention is narrow.

## Experiment 5

Experiments 1–3 indicate that the spatial extent of the attentional focus modulates the efficiency of attentional disengagement. Experiment 4 eliminates the possibility that the results stem from the temporal predictability of the target and subsequent readiness-to-respond effects. In Experiment 5, we address a further alternative account of the results. Specifically, due to their size difference, the small and large central squares will differ in saliency, with the larger square being more salient than the small square.^1^ Saliency may influence the efficiency of disengaging attention, with the consequence that the observed difference in the efficiency of disengaging attention in Experiments 1, 3, and 4 may stem from the difference in salience between the small and large central squares rather than from differences in the spatial extent of attention.

Although at first consideration this account of the results seems plausible, we believe it unlikely to account for the present results. Since high-salience items are known to capture attention (e.g., Theeuwes, 1991, 1992, 2010), disengaging attention would presumably be slower or more difficult from items that capture attention. On these grounds, since the large square is more salient than the small square, one would expect the disengagement of attention to be less efficient in the Large-Square condition. The results of Experiments 1, 3, and 4, however, show the exact opposite pattern of results, with attentional disengagement being *more* efficient in the Large-Square condition. Thus, an account based on low-level differences in saliency between the small and large central squares is not consistent with the results of Experiments 1, 3, and 4.

To confirm that differences in the salience of the small and large central squares are not the cause of the observed differences in the efficiency of disengaging attention, in Experiment 5 we equated the saliency of the central squares by increasing the thickness—and thus the saliency—of the line that forms the small central square. If saliency differences between the small and large central squares are the cause of the differences in the efficiency of disengagement observed in Experiments 1, 3, and 4, we should see no effect of central square size on disengagement in Experiment 5.

### Participants

Relying on the a priori power analysis reported in Experiment 4, a minimum sample of 36 participants was needed for an effect size of *ƞ*_p_^2^ = 0.133 to be detected with 90% probability with alpha set to 0.05.

Fifty-two undergraduate students participated in the experiment for course credit. Three participants were removed from analysis, one due to an equipment problem during the task, one due to reporting a possible diagnosis of ADD, and one due to their accuracy scores being more than three standard deviations below the mean. This left a total of 49 participants for analysis. 40 participants self-identified as female, 9 as male. 4 participants self-reported being left-handed, 1 ambidextrous, and 44 right-handed. The mean age of the participants was 21.26 years (SD = 6.08). All participants were naïve as to the purpose of the experiment, reported normal or corrected-to-normal vision, and provided written informed consent.

### Stimuli and procedures

The stimuli and procedures in Experiment 5 were the same as in Experiment 3 except that the thickness of the small central square was increased to make it more salient. The number of pixels forming the outline of the large square in Experiment 3 was four times greater than the number of pixels forming the outline of the small square. Thus, in Experiment 5, the thickness of the outline of the small square was increased four-fold (from 0.1° to 0.4°), thus equating the number of pixels forming the outlines of the small and large central squares.

### Results and discussion

**Fig. 8:**
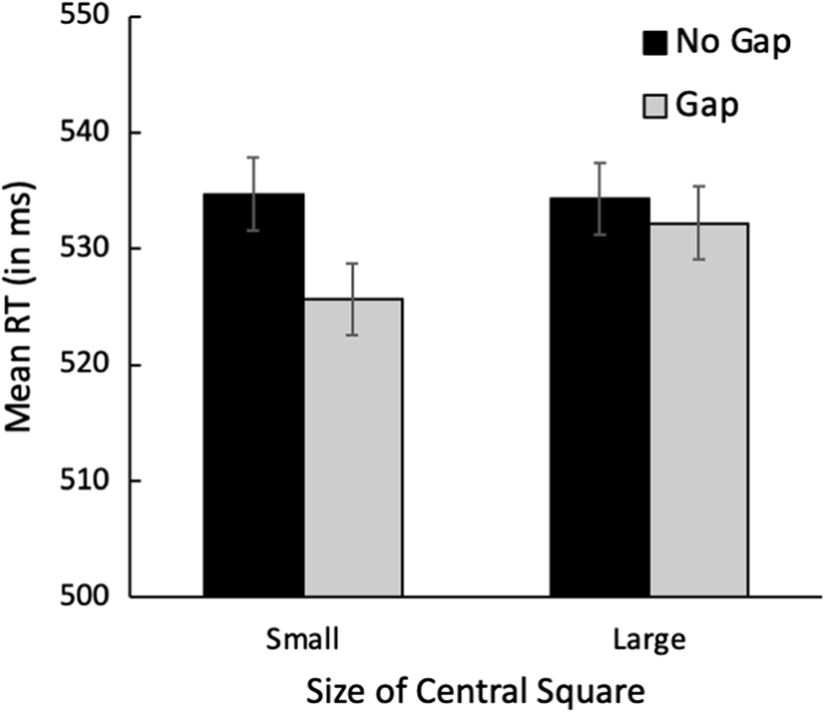
Mean RT in milliseconds as a function of central square size and gap presence. Error bars indicate ± one standard error of the mean

As in Experiments 1–4, trials with RTs ± 2 SD from the mean were excluded from analysis. Only trials on which the target was correctly identified were included for analysis. Mean RT as a function of the size of the abrupt-onset central square and the presence of a gap is illustrated in Fig. 8.

### Accuracy results

Reaction time is the primary measure of interest; however, as with the previous experiments, we first consider the accuracy data. Mean target identification accuracy was 97.2% (SD = 2.64) in the Small-Square Gap condition, 96.3% (SD = 2.96) in the Small-Square No-Gap condition, 96.2% (SD = 2.90) in the Large-Square Gap condition, and 96.9% (SD = 2.27) in the Large-Square No-Gap condition. The data were analyzed in a 2 (Central Square Size: Small, Large) × 2 (Gap Presence: No Gap, Gap) repeated-measures ANOVA. The main effects of Central Square Size and Gap were not significant (both *F* < 1). The interaction was significant, *F*(1, 48) = 8.17, *p* = 0.006, *ƞ*_p_^2^ = 0.145. In considering whether these results are evidence of a speed–accuracy trade-off, we note that the condition in which RTs are slowest (No-Gap, Small-Square condition) is associated with lower levels of accuracy; the condition in which RTs are fastest (Gap, Small-Square condition) is associated with higher levels of accuracy. Thus, there is no indication of a speed–accuracy trade off.

### Reaction time results

The data illustrated in Fig. 8 were analyzed in a 2 (Gap Presence: Gap, No Gap) × 2 (Size of central square: Small, Large) repeated-measures ANOVA. The analysis revealed a significant main effect of Square Size, *F*(1,48) = 4.25, *p* = 0.045, *ƞ*_p_^2^ = 0.081, and Gap Presence, *F*(1,48) = 10.16, *p* = 0.003, *ƞ*_p_^2^ = 0.175. Critically, the interaction between Gap Presence and Square Size was significant, *F*(1,48) = 4.76, *p* = 0.034, *ƞ*_p_^2^ = 0.090. These results closely replicate those of Experiment 3, with the significant interaction indicating that the magnitude of the gap effect is greater when the central square is small than when the central square is large. If the results of Experiments 1–4 stemmed primarily from differences in salience between the small and large central squares, no effect of square size should be present. The clear effect of central square size in Experiment 5 thus suggests that differences in the breadth of attention affect the efficiency of disengaging attention, with the disengagement of attention being less efficient when the focus of attention is narrow.

## General discussion

The present study examined whether the spatial extent of the attentional focus affects the efficiency at which it can be disengaged. In Experiment 1, we tested this by combining a gap paradigm to assess attentional disengagement with an abrupt-onset small or large central square to trigger reflexive attentional resizing. RTs were significantly faster in the Gap condition, in which the fixation cross was removed prior to the onset of the target, than in the No-Gap condition, in which the fixation cross remained present. The key finding was that the magnitude of the Gap/No-Gap difference was greater when attention was narrowly focused (Small-Square condition) than when it was broadly focused (Large-Square condition), a significant interaction which arose primarily from RT differences in the No-Gap condition. Unexpectedly, RTs also differed in the Gap condition, with RTs in the Small-Square condition being significantly faster than RTs in the Large-Square condition. The results of Experiment 2 suggest that this arises from faster processing of fixation offset in the Gap condition. Experiment 3 employed a modified paradigm that both minimized the likelihood of observers fixating a peripheral location and provided a replication of the results in Experiment 1. Experiment 4 eliminated the temporal predictability of the target’s onset and provided converging evidence to support the effect of focusing on the disengagement of attention by including a condition in which focusing could not occur. Experiment 5 confirmed that the results were not due to low-level saliency differences between the small and large central squares. Thus, the results of the present experiments strongly suggest that attention can be disengaged more efficiently when the attentional focus is large and less efficiently when the attentional focus is narrow.

It is worth considering whether generalized, nonspecific alerting might be responsible for the results of the present experiments. Nonspecific alerting serves to enhance the visual and response systems’ state of readiness when a signal is received that indicates the imminent onset of a target, but provides no specific information as to the target’s location. Alerting has been shown to result in both faster reaction times (e.g., Bernstein et al., 1970; Fernandez-Duque & Posner, 1997) and enhanced target identification accuracy (e.g., Jefferies et al., 2022; Nakayama & Mackeben, 1989; Spalek & Di Lollo, 2011). In the present Experiments 1 and 3, alerting could potentially arise from the onset or offset of the central square. However, since the onset and offset of the central square occurs in both the Gap and the No-Gap conditions, alerting effects cannot account for the differences in the magnitude of the gap effect (calculated as the RT difference between the No-Gap and the Gap conditions).

Of greater potential concern, alerting could also arise from the offset of the fixation cross, which occurs only in the Gap condition. Previous research has directly tested whether the gap effect is caused by alerting from the offset of the fixation cross. In their Experiment 3, Mackeben and Nakayama (1993) used two conditions to test whether it was the disappearance of the fixation cross or a general alerting or warning effect arising from the disappearance of fixation, that led to faster disengagement of attention in the Gap condition. In the first condition, Mackeben and Nakayama replaced the gap with a change to the fixation cross. Specifically, fixation changed from a plus sign to an ‘*x*’. This change to fixation would provide an alerting or warning signal, but would not trigger the disengagement of attention. In the second condition, the fixation cross did not change, but the entire background of the screen brightened for 33 ms. Again, this would provide an alerting or warning signal, but would not trigger the disengagement of attention. In both of these conditions, if alerting is the cause of the gap effect, then a gap effect should occur. On the other hand, if the cause is the removal of fixation, which triggers the disengagement of attention, then a gap effect should not occur. The results showed no gap effect in either condition, demonstrating that alerting triggered by the offset of fixation is not the cause of the gap effect. This is the case regardless of whether there is a relatively weak alerting signal (a change in fixation shape) or a relatively strong alerting signal (a brightening of the entire screen). As such, it is very unlikely that generalized altering can account for the results of the present set of experiments.

### Clinical implications

Given that the breadth of attention modulates the efficiency of disengagement, differences in the default breadth of attention may be the mechanism underlying individual and group differences in attentional disengagement. For example, many studies have shown that the focus of attention is unusually narrow in individuals with Autism Spectrum Disorder (ASD; e.g., Burack, 1994; Mann & Walker, 2003; Rincover & Ducharme, 1987; Robertson et al., 2013; Ronconi et al., 2013; Townsend & Courchesne, 1994). The present results suggest that this unusually narrow attentional focus might result in individuals with ASD having a delay in disengaging attention, making it more difficult for them to orient attention and switch from one task to another. This is quite consistent with their clinical symptoms, and individuals with ASD are characteristically hyperfocused, have narrow and restricted interests, and have difficulty switching between tasks (e.g., APA, 2013). Furthermore, there is some evidence that individuals with dyslexia have a narrow attentional focus (e.g., Moores et al., 2015, although see conflicting evidence by Facoetti et al., 2000 and Facoetti & Molteni, 2001). Given the present results, this narrow attentional focus would be disengaged more slowly, which may play a role in the atypical reading and scanning patterns exhibited by individuals with dyslexia. Likewise, individuals with schizophrenia seem to have an atypically narrow attentional focus (e.g., Elahipanah et al., 2011; Gray et al., 2014; Hahn et al., 2012; Leonard et al., 2017; but see Kopp et al., 1994), and the resulting delay in disengaging attention may play a role in their atypical processing of visual information. Finally, it has been shown that individuals high in anxiety disengage from feared stimuli more slowly than do individuals low in anxiety (e.g., Fox et al., 2001, 2002; Georgiou et al., 2005). Rather than being a direct result of their phobia, this delay in disengagement may stem from the fact that negative emotions lead to a narrower focus of attention (e.g., Derryberry & Tucker, 1994; Fredrickson, 1998).

In summary, the present results show that the efficiency of attentional disengagement is modulated by the breadth of the attentional focus—the disengagement of attention is delayed when the focus is small. In addition, the results underscore that attentional processes interact with one another, and that they must be considered not only separately, but in conjunction with one another.

## Data Availability

The data sets generated during and/or analysed during the current study are available from the corresponding author on reasonable request.

## Footnotes

1 We thank an anonymous reviewer for suggesting this possible account of the results and for suggesting Experiment 5.
